# Preprocedural computed tomography angiography-guided transcatheter arterial embolization for managing esophageal cancer bleeding: a case series study

**DOI:** 10.3389/fonc.2025.1598271

**Published:** 2025-10-07

**Authors:** Xue-Jiao Yang, Yong-Juan Wu, Jing-Zhong Wang, Yu-Lan Zheng, Guang-Yuan Cheng, Yun-Hua Cui, Xiao-Qi Li

**Affiliations:** ^1^ Department of Respiratory and Critical Care Medicine, Xiangyang Central Hospital, Affiliated Hospital of Hubei University of Arts and Science, Xiangyang, China; ^2^ Department of Interventional Radiology, Xiangyang Central Hospital, Affiliated Hospital of Hubei University of Arts and Science, Xiangyang, China

**Keywords:** CT angiography, transcatheter arterial embolization, esophageal cancer bleeding, arterial-esophageal fistula, interventional radiology, case series study

## Abstract

**Objective:**

While endoscopy is the first-line treatment for non-variceal upper gastrointestinal hemorrhage, its role in managing upper gastrointestinal tumor bleeding is limited. This study aims to evaluate the effect of computed tomography angiography (CTA)-guided transcatheter arterial embolization (TAE) in achieving hemostasis for esophageal cancer bleeding.

**Methods:**

A retrospective case series was conducted at our hospital, involving eight patients who underwent preprocedural CTA-guided TAE for esophageal cancer bleeding between August 2023 and August 2024.

**Results:**

Of the eight patients (seven males, mean age 67 ± 12.9 years) who underwent CTA-guided TAE for esophageal cancer bleeding, seven achieved both technical and clinical success. One patient failed to achieve embolization due to missed identification of a pseudoaneurysm in the superior thyroid artery, resulting in death from hemorrhage. Two patients experienced mild chest or neck discomfort, which resolved with conservative management. During follow-up, five patients experienced recurrent hemorrhage, with four dying and one successfully treated with a second TAE. One of the remaining two patients died from disease progression, and the other remained recurrence-free for 270 days.

**Conclusion:**

CTA-guided TAE demonstrates high technical and clinical success rates for achieving hemostasis in esophageal cancer bleeding with mild and manageable complications.

## Introduction

Esophageal cancer bleeding is one of the most life-threatening acute complications in esophageal cancer patients, often presenting as melena or hematemesis (1, 2). Bleeding may result from tumor ulceration or the formation of an arterial-esophageal fistula (AEF), a condition in which tumor invasion or treatment erodes adjacent arterial walls, creating an abnormal passage between the artery and esophagus that can result in massive hemorrhage (3). In particular, AEF is associated with a high mortality rate ranging from 46.6% to 63% and requires urgent thoracic endovascular aortic repair or surgical intervention (2, 4–6). Currently, upper gastrointestinal endoscopy is the first-line treatment for non-variceal upper gastrointestinal hemorrhage (7, 8). Nevertheless, no available endoscopic treatment demonstrates long-term efficacy (9) and approximately 10% of patients experience rebleeding after endoscopic treatment, with reduced long-term hemostasis success and increased mortality (10). Additionally, endoscopy poses a risk of esophageal perforation (11), underscoring the need for alternative treatment options.

Transcatheter arterial embolization (TAE) has emerged as an effective alternative to endoscopic treatment (12–14), particularly for non-variceal upper gastrointestinal bleeding that is unmanageable with endoscopy (15). However, the vascular anatomy associated with esophageal cancer bleeding is complex, involving both tumor-feeding arteries and potential AEFs. Thus, accurately identifying bleeding vessels is crucial for TAE success. With advancements in imaging and interventional techniques, preprocedural computed tomography angiography (CTA) has become a valuable tool for evaluating tumor extent and identifying bleeding-related arteries, providing crucial anatomical guidance for TAE (16–18). This study aimed to evaluate the therapeutic effect of CTA-guided TAE for esophageal cancer bleeding.

## Methods

### Study design

This is a case series study performed at our hospital, between August 2023 to August 2024, involving patients with esophageal squamous cell carcinoma who presented with upper gastrointestinal bleeding and underwent CTA-guided TAE after unsuccessful or unsuitable endoscopic treatment. The inclusion criteria included: 1) had prior pathological diagnosis of esophageal squamous cell carcinoma and presented with varying degrees of hematemesis; 2) underwent unsuccessful endoscopic hemostasis or were deemed unsuitable for endoscopic hemostasis; 3) received conservative medical treatments (e.g., proton pump inhibitors and transfusion support) without achieving bleeding control; and 4) received preprocedural CTA-guided TAE for treating upper gastrointestinal bleeding. Informed consent was waived due to the retrospective nature of the study. Ethical approval was granted by the ethics committee (No. 2024-177), and the study adhered to the principles of the Declaration of Helsinki.

### Data collection

The CTA images were collected for all patients, showing (1) the primary tumor and its extent of invasion; (2) the anatomical structures of potential tumor-feeding arteries, including the superior thyroid artery, inferior thyroid artery, bronchial artery, proper esophageal artery, posterior intercostal artery, left gastric artery, and inferior phrenic artery; and (3) the presence or absence of an AEF. The diagnostic criteria for AEF included pseudoaneurysm protruding into the esophageal lumen and intrathrombus air bubbles, and extravasation of contrast material into the esophagus. AEFs were further categorized into aortic AEFs and non-aortic AEFs.

Follow-up data, including patient survival status, time to death or last follow-up, cause of death, recurrence of bleeding, need for re-intervention, and procedure-related complications, were collected through the hospital’s electronic medical record system.

### CTA-guided TAE

All patients underwent a dedicated preprocedural CTA in the diagnostic CT suite using a Siemens SOMATOM Drive dual-source CT scanner. Since initial presentation CT scans may not be sufficient for interventional planning, this dedicated CTA was performed to provide detailed vascular mapping. The scanning range extended from the neck down to the lower edge of the first lumbar vertebra, ensuring visualization of the entire esophagus and possibly affected arterial vessels. A threshold-trigger technique was used for intravenous contrast injection, with the trigger layer positioned at the origin of the descending aorta and a threshold set at 100 HU. After scanning, high-resolution image reconstruction (slice thickness 1 mm, interval 1 mm) was performed based on lesion extent. For lesions with multiple possible feeding arteries, all relevant vasculature suggested by CTA was interrogated with selective angiography. In cases where CTA did not clearly demonstrate a vascular abnormality, selective imaging of possible culprit vessels was still performed to avoid missed diagnosis. Two radiologists with at least five years of experience analyzed the images to ensure consistency and accuracy.

A 5F vascular sheath was inserted through the right femoral artery, and a 5F angiographic catheter was used for catheter angiography. Based on CTA findings, selective angiography was performed on the bronchial artery, proper esophageal artery, branches of the thyrocervical trunk (e.g., inferior thyroid artery), superior thyroid artery, posterior intercostal artery, celiac artery, and subclavian artery, as required. If angiography identified active bleeding (contrast media extravasation), pseudoaneurysm, abrupt vascular cutoff, or marked tumor staining, a 1.98F microcatheter (ASAHI INTECC, ASAHI Masters PARKWAY SOFT) and a microwire (ASAHI INTECC, Streaming 14) were advanced distally for targeted embolization. Appropriate embolic materials were then deployed at the operator’s discretion. In patients with contrast media extravasation, pseudoaneurysm, or abrupt cutoff, both particles [polyvinyl alcohol (PVA) or gelatin sponge] and coils were used, whereas in patients with only tumor staining, particles (PVA and/or gelatin sponge) were deployed. The endpoint of embolization was defined as the complete cessation of blood flow in the identified culprit bleeding artery.

Technical success was defined as the successful embolization of all possible culprit major arteries. Clinical success was defined as the absence of active bleeding and improvement in clinical symptoms (stable hemoglobin levels) within 3 days after TAE. Major periprocedural and postoperative complications were classified according to Society of Interventional Radiology practice guidelines, with severe complications requiring further treatment or extended hospitalization and mild complications resolving spontaneously (19).

### Statistical analysis

Only descriptive analysis was performed. The analysis was conducted using SPSS software (version 23.0; IBM Corp., Armonk, NY, USA).

## Results

A total of eight patients with esophageal squamous cell carcinoma who experienced upper gastrointestinal bleeding were included in the analysis. The cohort consisted of seven males and one female, with a mean age of 67 ± 12.9 years. Six patients had received prior antitumor therapy, including chemotherapy, chemoradiotherapy, immunotherapy, or radiotherapy. The primary tumors were located in the cervical, upper, middle, or lower esophagus, with varying TNM stages. CTA identified tumor-related vascular abnormalities in several cases, such as AEFs. No cases of aortic arterioesophageal fistulas were identified in this cohort. Detailed patient demographics, clinical characteristics, CTA findings, embolic agents, and TAE outcomes are summarized in **Table 1**.

**Table 1:** Patient characteristics and embolization details.

N./Age/Sex	TNM stage	Esophageal level	Antitumor therapy	CTA findings	TAE					Remark
					Angiographic findings	Embolic agent	Technical success	Clinical success	Complications	
1/58/M	cT2N1M0	Cervical/Upper	Chemotherapy/Immunotherapy	–	RIBA ITA Esophageal branch of the TT	PVA+GSP	Yes	Yes	neck discomfort	died due to recurrence 42 days later
2/49/M	cT4NxM0	Middle	Chemotherapy	BAPA	RIBA PEA BAPA	PVA+GSP+Coils	Yes	Yes	No	died due to recurrence 63 days later
3/59/M	cT4N3M0	Cervical	Chemoradiotherapy/Immunotherapy	STA pseudoaneurysm	ITA*	–	No	No	–	death
4/84/M	cT4NxM0	Middle	Radiotherapy	BAPA	BAPA PEA	GSP+Coils	Yes	Yes	No	died due to recurrence 85 days later
5/79/M	cT4NxMx	Upper/Middle	Chemotherapy/Immunotherapy	BAPA	BAPA BA	GSP+Coils	Yes	Yes	No	Died due to disease progression 103 days later
6/69/M	cT4NxM0	Middle/Lower	No	–	RIBA PEA	PVA+GSP	Yes	Yes	chest discomfort	270 days without recurrence
7/58/M	cT4N1M1	Upper/Middle	Chemoradiotherapy/Immunotherapy	–	RIBA LGA PEA	GSP	Yes	Yes	No	died due to recurrence 133 days later
8/80/F	cT4NxM1	Middle/Lower	No	–	LGA LHA PIA IPA PEA	GSP	Yes	Yes	No	recurrence 396 days later/TAE achieved clinical success

*: Selective angiography of bilateral superior thyroid arteries was not performed; TAE, transcatheter endovascular embolization; BAPA, bronchial artery pseudoaneurysm; STA, superior thyroid artery; ITA, inferior thyroid artery; BA, bronchial artery; RIBA, right intercostal bronchial artery; PEA, proper esophageal artery; LGA, left gastric artery; LHA, left hepatic artery; IPA, inferior phrenic artery; PIA, posterior intercostal artery; TT, thyrocervical trunk; PVA, polyvinyl alcohol; GSP, gelatin sponge particle.

**Figure 1:**
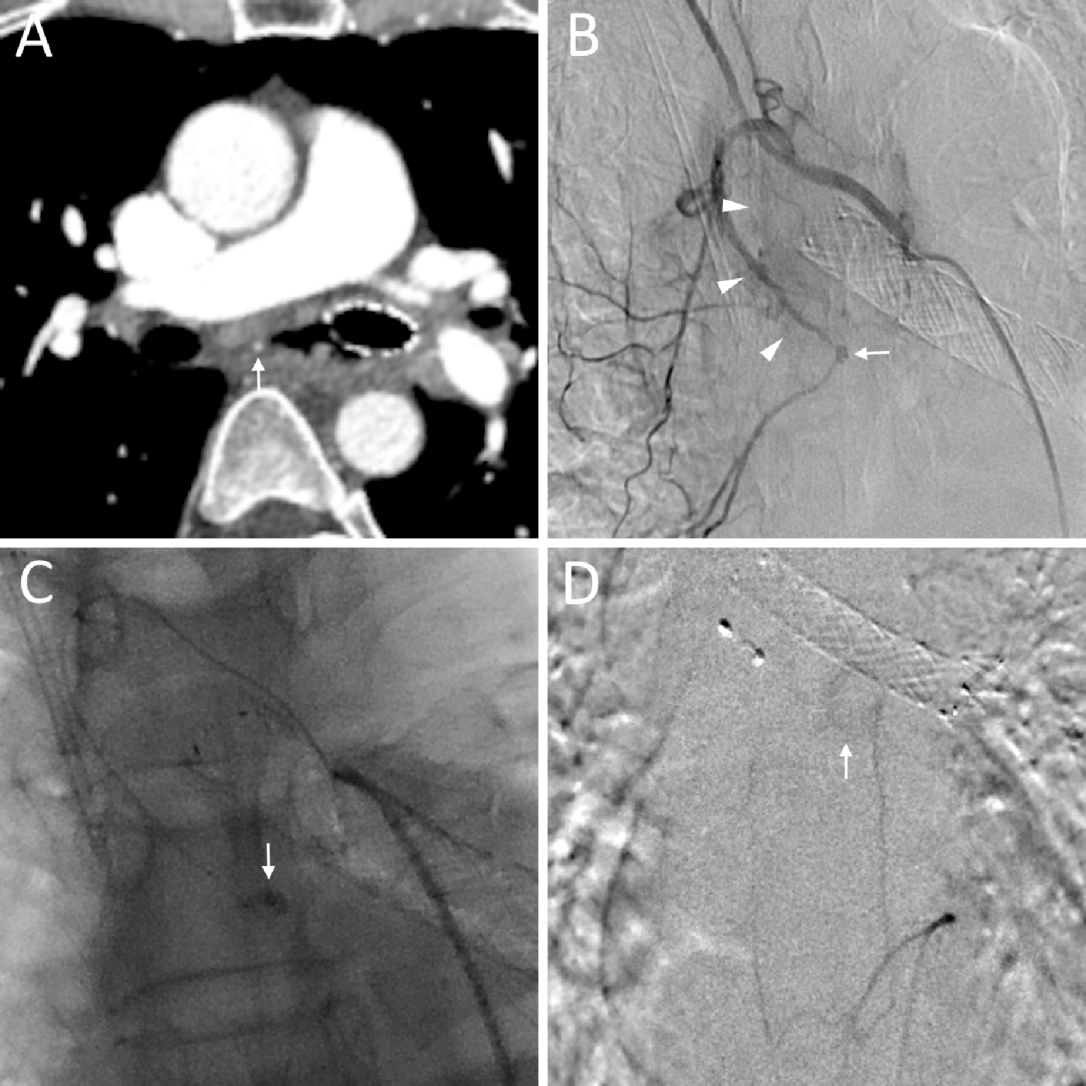
A 49-year-old male (No. 2) diagnosed with esophageal cancer presented with hematemesis. **(A)** Transverse CT showing a bronchial artery pseudoaneurysm (arrow) in the tumor area; **(B)** Right intercostal bronchial artery angiography showing the bronchial artery pseudoaneurysm (arrow) and tumor staining (triangular arrows); **(C)** The microcatheter was super-selectively advanced into the bronchial artery, and manual contrast injection revealed a ruptured pseudoaneurysm with active bleeding (arrow). **(D)** Proper esophageal artery angiography showing tumor staining (arrow).

**Figure 2:**
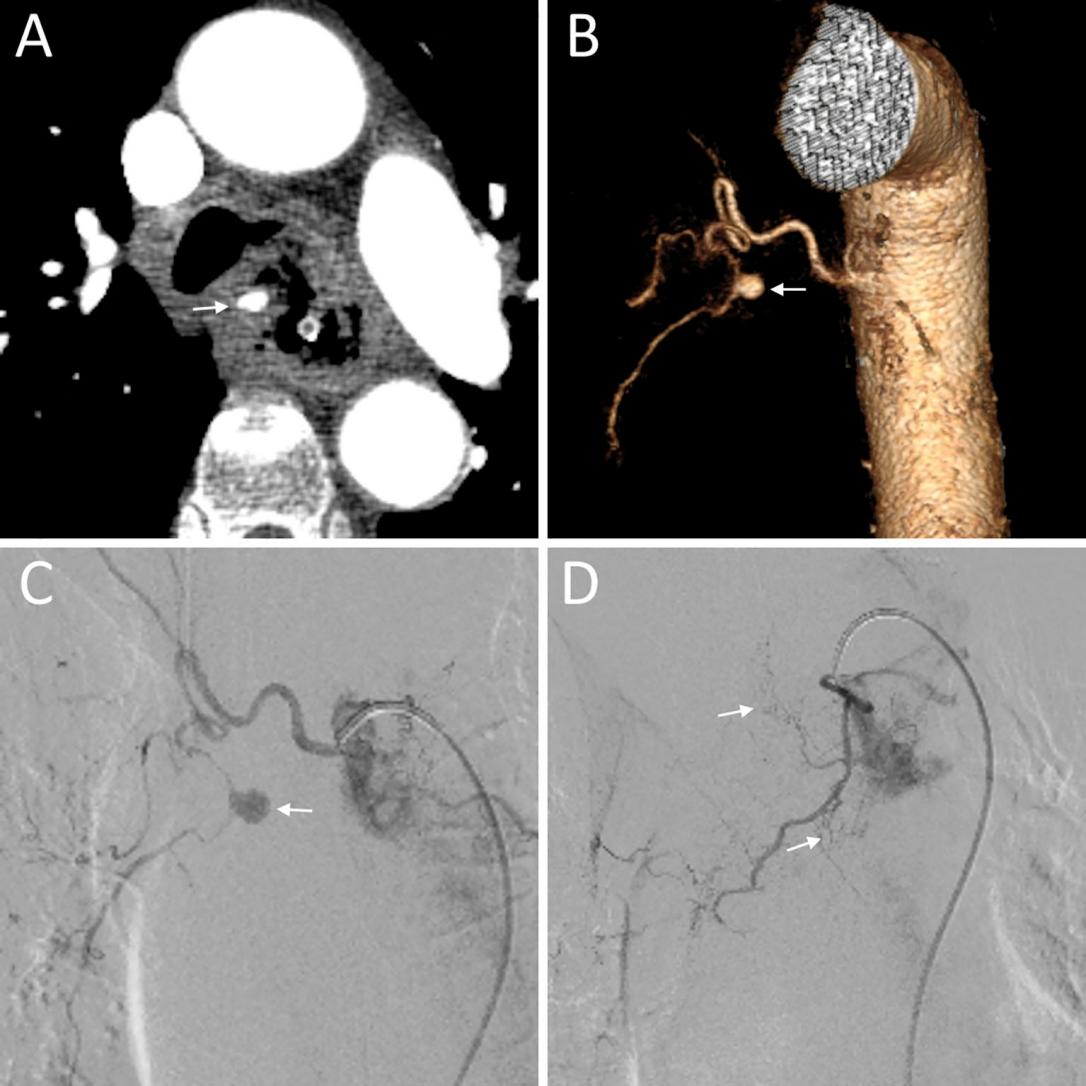
A 79-year-old male (No. 5) diagnosed with esophageal cancer presented with hematemesis. **(A)** Transverse CT showing a bronchial artery pseudoaneurysm (arrow) in the tumor area; **(B)** Volume-rendered reconstruction displaying the bronchial artery pseudoaneurysm (arrow); **(C)** Right intercostal bronchial artery showing the bronchial artery pseudoaneurysm (arrow); **(D)** Angiography of the bronchial artery demonstrating tumor staining (arrows).

**Figure 3:**
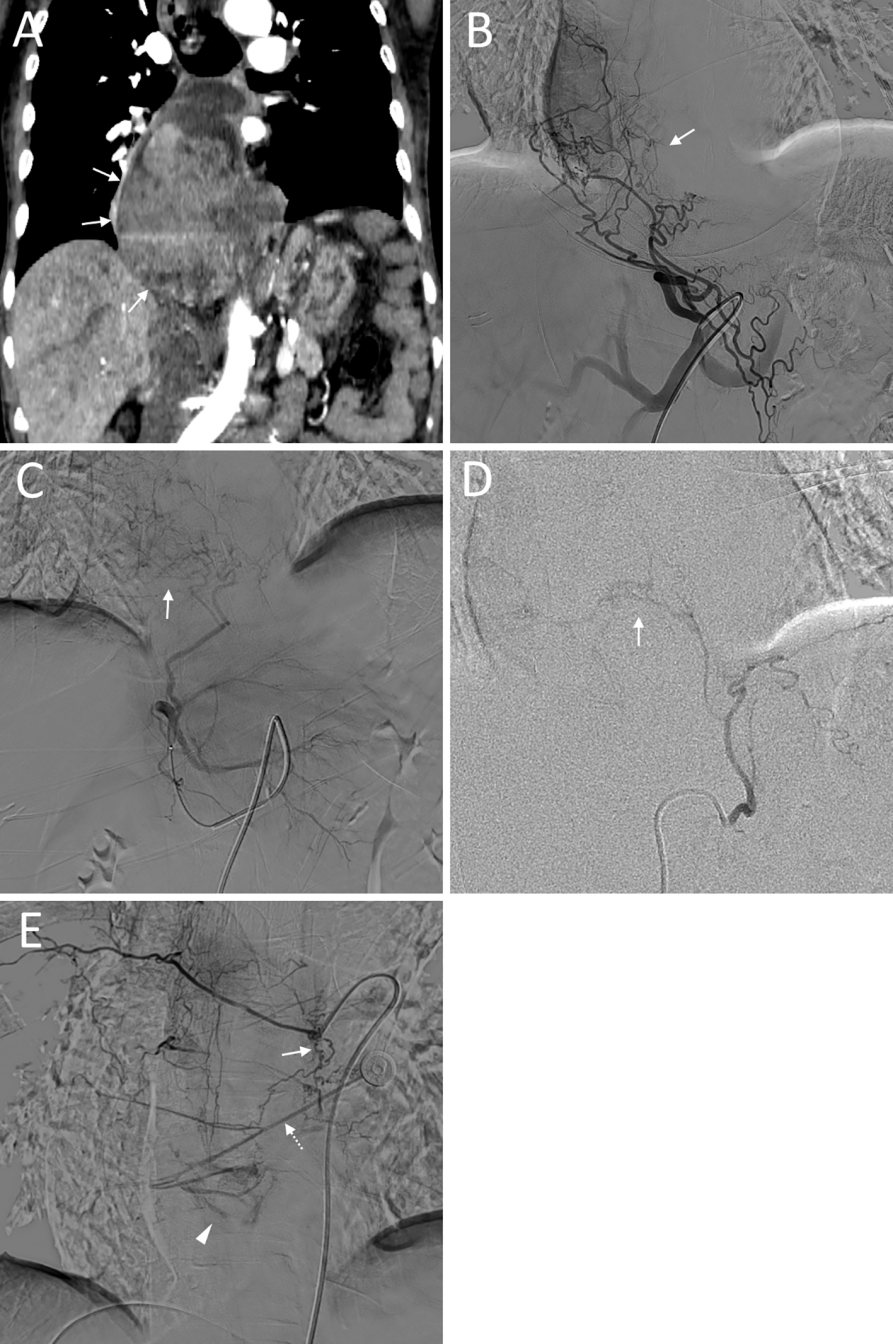
An 80-year-old female (No. 8) diagnosed with esophageal cancer presented with hematemesis. **(A)**: Coronal CT showing the tumor (arrow); **(B)**: Angiography of the left gastric artery displaying tumor staining (arrow); **(C)**: Angiography of the left hepatic artery displaying tumor staining (arrow); **(D)**: Angiography of the left inferior diaphragmatic artery displaying tumor staining (arrow); **(E)**: Angiography of the right posterior seventh intercostal artery showing branching (arrow) communicating with the proper esophageal artery (dashed arrow), jointly participating in tumor staining (triangular arrow).

Guided by preprocedural CTA, the interventional radiologists located the likely culprit bleeding arteries and performed angiography and embolization. Preprocedural CTA identified three patients with non-aortic AEFs, manifesting as bronchial artery pseudoaneurysm (No. 2, **Figure 1**; No. 4, and No. 5, **Figure 2**) and superior thyroid artery pseudoaneurysm (No. 3). Angiography could further identify bleeding from multiple arteries that led to tumor staining, such as the left gastric artery, left hepatic artery, inferior phrenic artery, proper esophageal artery and posterior intercostal artery (No. 8, **Figure 3**). Seven of the eight patients achieved both technical success and clinical success following TAE. One patient failed to achieve embolization due to complex vascular anatomy and missed detection of a pseudoaneurysm in the left superior thyroid artery, resulting in death from hemorrhage (No. 3). Two patients experienced mild complications, including neck (No. 1) and chest discomfort (No. 6), which resolved spontaneously with conservative management.

During follow-up, five patients had rebleeding, four of whom died due to the recurrent hemorrhage (No. 1, 2, 4, 7), while one received a second TAE that successfully controlled the bleeding (No. 8). One patient died from tumor progression (No. 5), and one patient had no further episodes of bleeding (No. 6).

## Discussion

In this case series study, we reported that preprocedural CTA-guided TAE achieved technical success and clinical success in seven of eight patients with esophageal cancer bleeding, whereas only two patients experienced mild complications. These findings suggest that TAE may be a promising treatment option for esophageal cancer bleeding.

While endoscopic hemostasis remains the primary treatment for non-variceal upper gastrointestinal bleeding, its role is limited in initial bleeding control and rebleeding prevention, and lacks long-term efficacy (7, 9). Furthermore, evidence suggests that ‘emergent’ endoscopy (within 6 hours from the time of patient presentation) is associated with a higher in-hospital rate and 30-day mortality, especially in hemodynamically unstable patients (20); whereas ‘urgent’ endoscopy (within 12 hours from the time of patient presentation) could not prevent further bleeding or reduce mortality rate (21). In contrast, TAE offers a potential alternative for rapid hemostasis, while reducing blood transfusion requirements and avoiding complications associated with emergency surgery in patients who have failed endoscopic treatment or are unsuitable for endoscopy. Previous reports show that early clinical success rates for TAE in non-variceal upper gastrointestinal bleeding range from 44% to 94% (22). For arterial bleeding from esophageal lesions, early TAE clinical success rates are reported to be 77.8%-100% (12, 14, 23), aligning with the success rate observed in our study.

Esophageal cancer bleeding is often complex, with approximately 53.8% of cases due to tumor ulceration and 12.0% related to AEF. In tumor ulcer bleeding, the culprit bleeding vessel is typically the tumor-feeding artery; however, the tumor blood supply may be segmental and have multiple resources. Depending on the location of the tumor, feeding arteries may include the superior or inferior thyroid artery, bronchial artery, proper esophageal artery, posterior intercostal artery, left gastric artery, inferior phrenic artery, short gastric artery, or left hepatic artery, with a high degree of variability especially noted in the bronchial arteries (12, 24, 25). With the spread of the tumor, abnormal anastomoses can appear, further increasing both the number and variability of culprit blood vessels. Meanwhile, AEFs can be categorized into aortic AEF and non-aortic AEF (26), which can be managed with endovascular stent-graft placement and TAE, respectively (4). These complex, yet clinically important, variabilities in esophageal cancer bleeding underscore the need for accurate and comprehensive identification of culprit arteries and potential AEFs, thereby guiding interventional radiologists in devising intervention approaches and in choosing appropriate embolic materials (27, 28), ultimately improving the likelihood of successful hemostasis while minimizing complications. In addition, our findings suggest that recurrent bleeding may not be solely determined by the choice of embolic agents: the two patients treated with gelatin sponge alone experienced very late recurrence well beyond the absorption period, whereas three others treated with gelatin sponge combined with permanent agents still had early recurrence. Notably, patients without prior antitumor therapy tended to have longer recurrence-free intervals, suggesting that tumor necrosis and ulceration following such therapy may play a more important role in rebleeding than the use of temporary versus permanent embolic agents. However, this observation requires further validation in larger studies.

This study has several limitations. Firstly, the small sample size of only eight patients and the single-center design limits the generalizability of the findings and reduces the statistical power to draw robust conclusions. Secondly, the absence of a control group makes it difficult to directly compare CTA-guided TAE with alternative treatments, such as surgery or other endovascular interventions, thereby limiting the ability to evaluate its relative efficacy. Thirdly, as four out of eight patients died from rebleeding, the long-term effectiveness of TAE in preventing recurrent hemorrhage remains uncertain and warrants further investigation. Addressing these limitations in future research, such as conducting multi-center, prospective studies with larger cohorts and comparative analyses, would provide stronger evidence to support the use of this intervention in clinical practice.

## Conclusion

CTA-guided TAE is a potentially effective option for achieving hemostasis in esophageal cancer bleeding, with a high rate of technical and clinical success and mild, manageable complications observed in this case series. Further studies with larger cohorts and longer follow-up periods are needed to evaluate its long-term efficacy and role in preventing recurrent bleeding.

## Data Availability

The raw data supporting the conclusions of this article will be made available by the authors, without undue reservation.
